# Light quality and quantity affect graft union formation of tomato plants

**DOI:** 10.1038/s41598-021-88971-5

**Published:** 2021-05-10

**Authors:** Ahmed Fathy Yousef, Muhammad Moaaz Ali, Hafiz Muhammad Rizwan, Ahmed Gomaa Gad, Dangdi Liang, Li Binqi, Hazem M. kalaji, Jacek Wróbel, Yong Xu, Faxing Chen

**Affiliations:** 1 College of Horticulture, Fujian Agriculture and Forestry University, Fuzhou, 350002 China; 2 Department of Horticulture, College of Agriculture, University of Al-Azhar (Branch Assiut), Assiut, 71524 Egypt; 3 Plant Pathology Department, Faculty of Agriculture, Alexandria University, El-Shatby, Alexandria, 21545 Egypt; 4 Department of Plant Physiology, Institute of Biology, Warsaw, University of Life Sciences SGGW, 159 Nowoursynowska 159, 02-776 Warsaw, Poland; 5 Department of Bioengineering, West Pomeranian University of Technology in Szczecin, 17 Słowackiego Street, 71-434 Szczecin, Poland; 6 College of Mechanical and Electronic Engineering, Fujian Agriculture and Forestry University, Fuzhou, 350002 China; 7 Institute of Machine Learning and Intelligent Science, Fujian University of Technology, 33 Xuefu South Road, Fuzhou, 350118 China

**Keywords:** Cell biology, Developmental biology, Physiology, Plant sciences, Environmental sciences

## Abstract

It is already known that there are many factors responsible for the successful formation of a graft union. However, the role of light has been little studied. In an anatomical study, Scanning Electronic Microscope (SEM) was used to explore the effects of different light-emitting diodes (LEDs) on graft union formation in grafted tomato. In addition, the expression genes related to Auxin hormone signaling pathway (SAUR67, AUX1, ARF30, and LAX3) was investigated. The obtained results showed that the concrescence process occurred faster under R7:B3 light conditions, as compared to blue (B) and white fluorescent (WFL) lights. Red light application caused a delay in the vascular tissue differentiation, which may lead to callus development on both sides, causing junctional failure and resulting in ineffective graft junctional arrangement. The expression of genes related to Auxin hormone significantly increased by R7:B3 application. We suggest that LED spectra affects the graft development of tomato plants and can improve the performance of grafted tomato seedlings.

## Introduction

Vegetable grafting is a popular method for improving plant health against biotic and abiotic factors and is used in many countries^1,2^. Successful grafting requires specific environmental conditions during the acclimatization process (matrix healing). Great precision is required by skilled workers during the grafting process. Proper acclimatization is crucial for the survival of grafted plants^3^.

To ensure good healing and acclimatization of the grafting matrix, control of the microclimate of the grafted plants is essential. Previously, the traditional method of shading with plastic or organic fibers was used to lower the temperature and increase the relative humidity around the plants until grafting is successful^4^. Although it is very difficult to control the environmental conditions during grafting of plants under normal conditions. Many countries such as China, Japan and Korea have developed acclimatization chambers for better growth of union matrix and successful grafting of vegetables. Some researchers have reported rapid growth, good survival rate and remarkable quality of seedlings grown in cure and acclimation chambers^5–7^. Others, reported that, some plant hormones, e.g. auxins, are involved in the growth of plant vascular tissue by promoting cell division, elongation and development, and can be synthesized in the plant body^8^.

Light is a unique abiotic factor involved in the lateral transport of hormones (auxins) within the plant body^9^. Plant grafting is mainly influenced by the availability of hormones such as auxins and cytokinins^9^. The quality and intensity of light radiation can also affect the availability of auxins^10,11^. During the grafting process, scion usually suffers from water deficiency due to excessive evaporation. Proline—an amino acid, acts as an osmoregulatory agent and prevents water loss from the plant. Under drought stress and exposure to various light spectra, the plant produces carbohydrates, proline and auxins which help to resist abiotic stress^12^. Photosynthetically active radiation (PAR) can affect the availability of carbohydrates—the main source of energy. Excessive amount of carbohydrates can affect cell division and elongation, which affects the healing and acclimation of the composite matrix during the grafting process. Almansa et al.^13^ reported that the grafting ability of macadamia was affected by the amount of carbohydrates in the scion.

Plant hormone signal transduction is associated with grafting^14,15^. The plant hormones play an important role in the reconnection of vascular bundles during the grafting process, as reviewed by Nanda and Melnyk^16^. Early studies of graft morphology showed that the reconnection of vascular bundles between the rootstock and the scion is an important feature of graft healing affinity^17^. Light emitting diodes (LEDs) are a suitable light source because they have long lifetime, durability, portability and wavelength suitable for the target. Therefore, it is expected that LEDs should be used as a good and meaningful light source for planting under controlled atmosphere. Several studies showed the effects of LED on tomato growth and development, such as morphogenesis, chlorophyll content, photochemistry, leaf anatomy and photosynthesis^18–22^. During drought stress, the synthesis of carbohydrates, proline, and auxins is affected by light intensity and spectrum^23^. For this reason, artificial light (LEDs) is used to improve the grafting process of vegetables^24^. For the successful grafting of tomato, the LEDs can be used as a light source and the light intensity can be easily controlled^25^. In particular, the area efficiency of the space of the healing chambers is significantly increased by using vertical surfaces.

Scanning Electronic Microscope (SEM) technique is the best approach for observing the early stages of the grafting process and the structural changes that occur between the rootstock and the scion, thus increasing the understanding of the formation of the graft union^26,27^.

In the present study, we characterized the effects of LEDs on the development of graft union structure in tomato seedlings by analysing the morphological anatomy at different stages and examining the gene expression performance of genes related to the Auxin hormone signaling pathway [Small Auxin up RNA (SAUR67), Auxin transporter protein (AUX1), Auxin Response Factor (ARF30), Auxin transporter-like protein (LAX3)] under different optical spectra.

## Results

**Figure 1:**
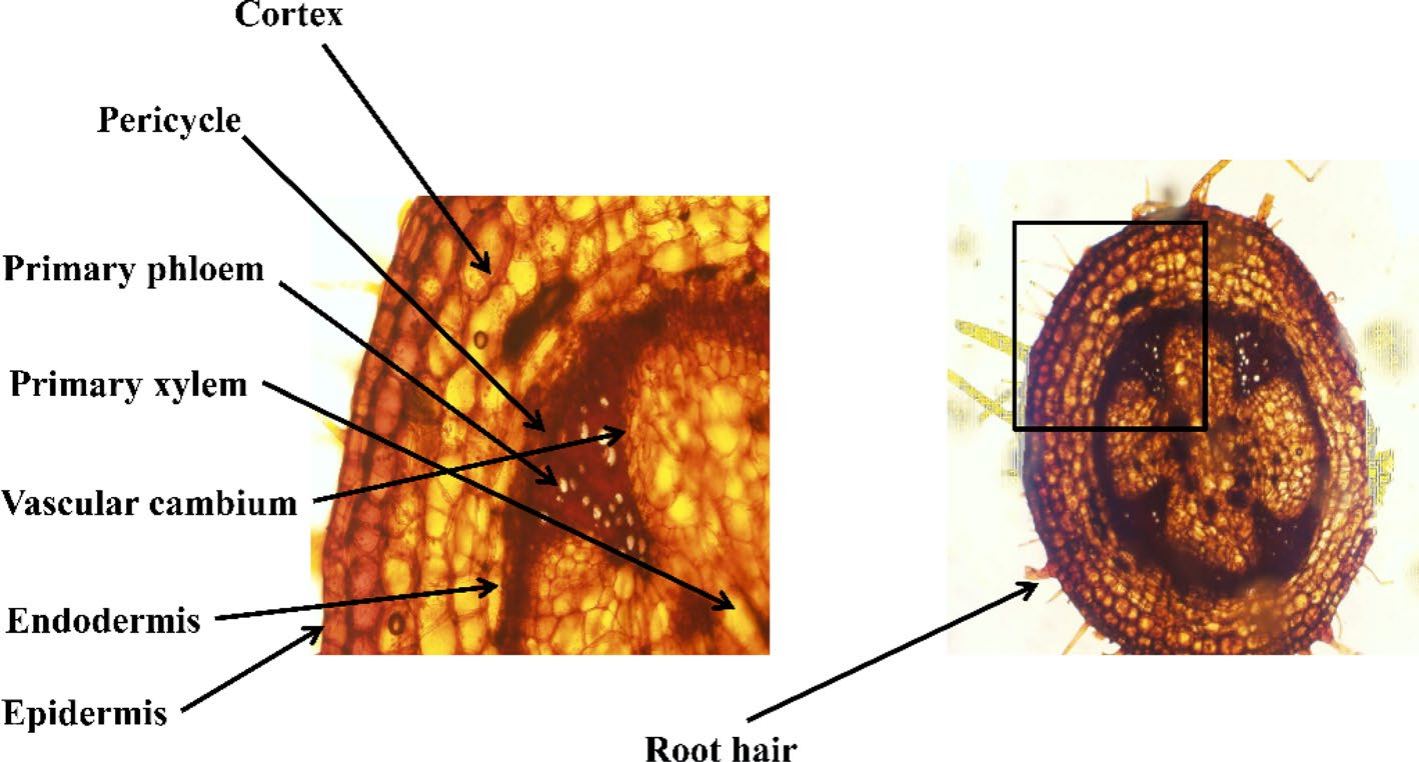
Structure of the tomato stem made by freehand section using paraffin sectioning technique.

Tomatoes share vascular properties with other plants. To ensure the safety of the inner tissues, the upper layer (epidermis) has the same function as the skin of animals. Cortex, phloem, xylem, and the core or pith are other tissue layers located under the epidermis. Each tissue has its specific functions and properties. The vascular cambium is the separating layer between each tissue- xylem and phloem transport food material within the plant body. The new tissues are formed, and the vascular cambium is found as shown in Fig. 1. The inner part contains spongy tissue called core or pith, which serves as the stem's food storage.

### The anatomical structure of the graft union at 5 DAG

**Figure 2:**
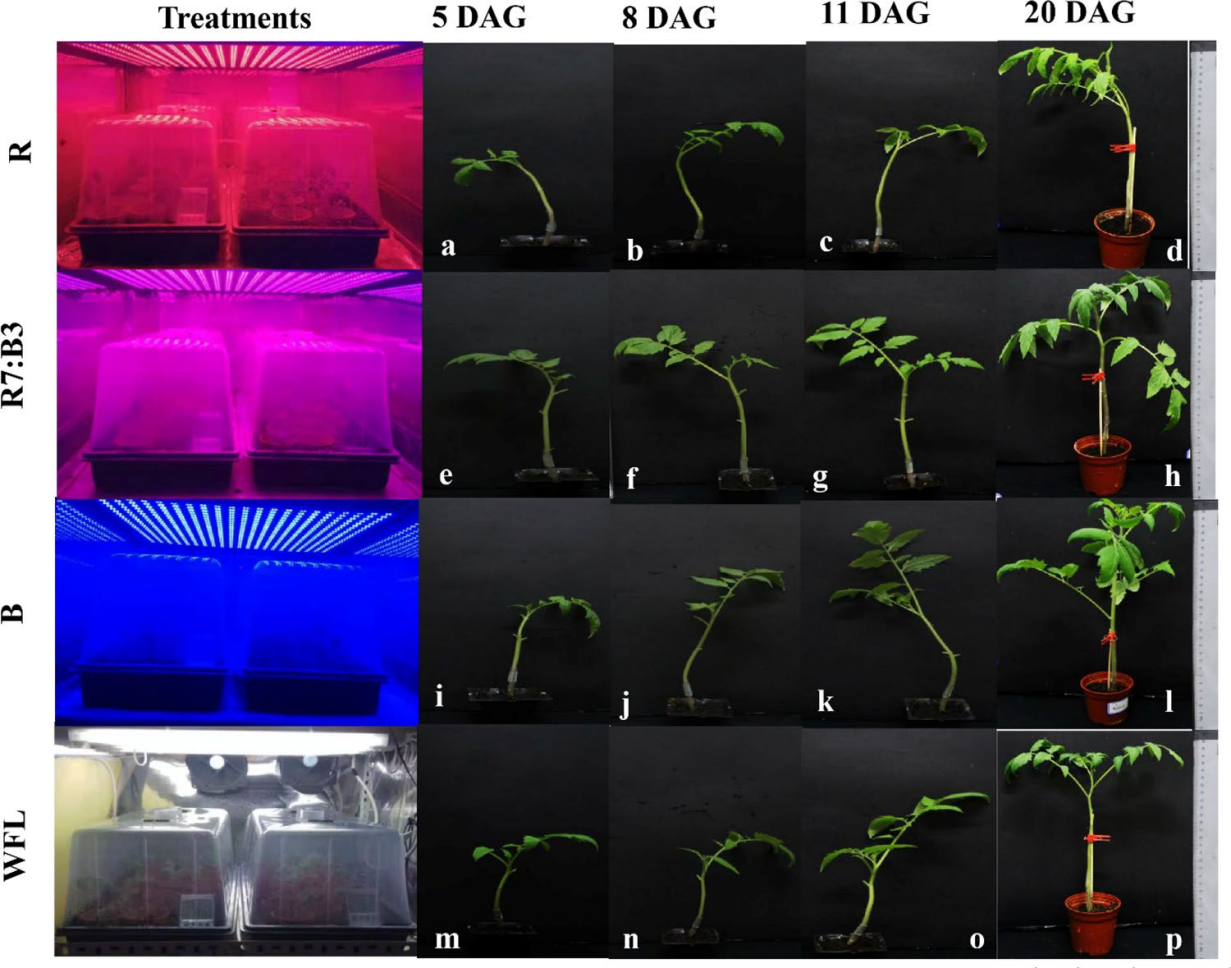
The stages of grafted seedlings in which the SEM examination was performed on the 5th, 8th, 11th and 20th days after grafting (DAG).

**Figure 3:**
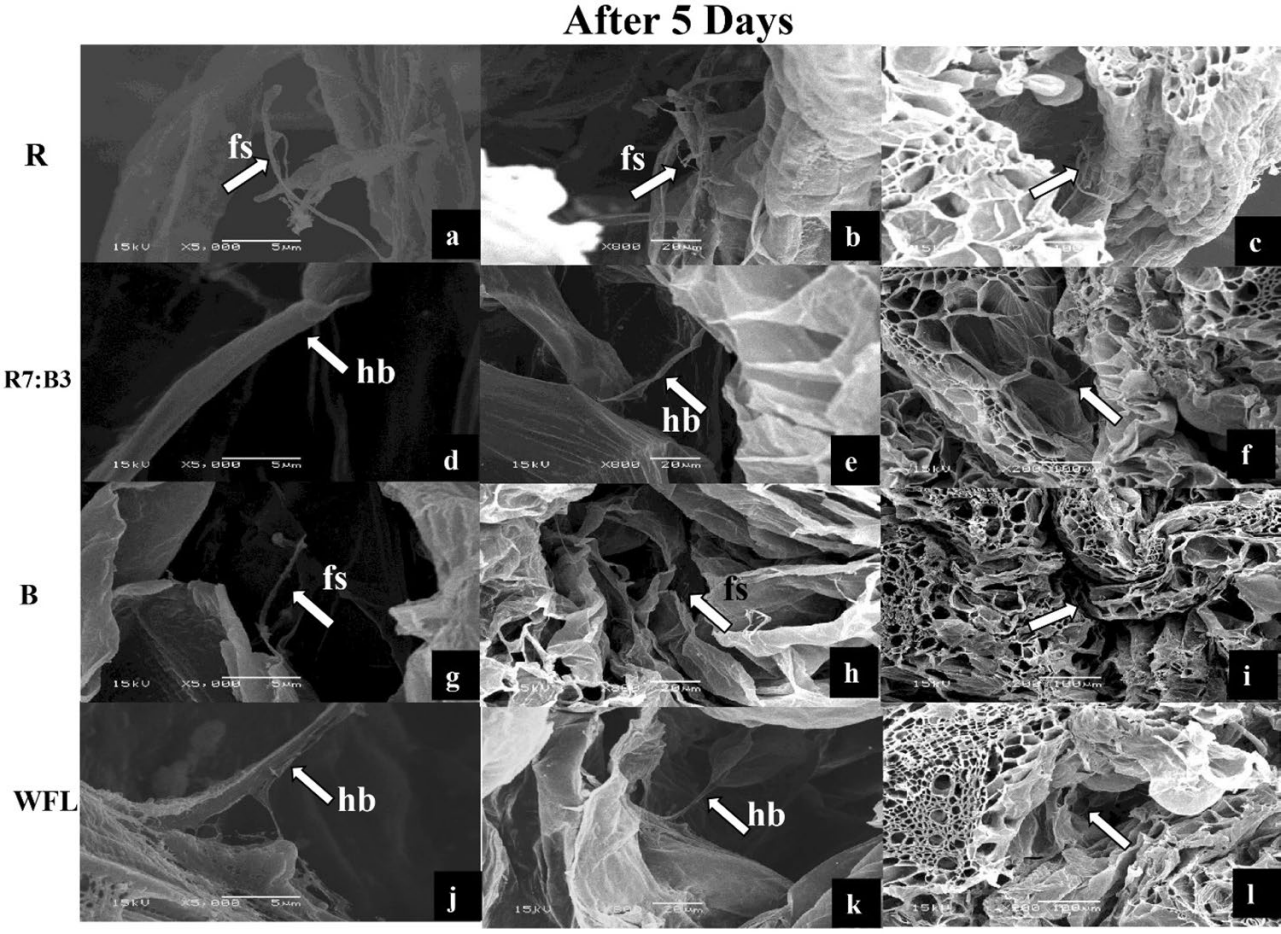
SEM images of interconnections between rootstock and scion after 5 days under R light (**a**–**c**), R7:B3 light (**d**–**f**), B light (**g**–**i**), and WFL light (**j**–**l**) light at the 5th days after grafting. fs—filamentous structures; hb—horizontal bridge.

During the first term of grafting (5 DAG) (Fig. 2a,e,i,m), some filamentous structures (fs) between scion and rootstock were observed in the SEM images before the horizontal bridge (hb) and callus formation (cf), indicating the first contact between the scion and rootstock under R (Fig. 3a–c) and blue light (Fig. 3g–i). It was the first contact between scion and rootstock under R and blue light, but had not yet formed new phloem and xylem elements between scion and rootstock. While under R7:B3 (Fig. 2d–f) and WFL (Fig. 2j–l) some horizontal bridges (hb) were observed connecting transverse structures and favouring between scion and rootstock. It is noticeable that these secreted filamentous structures (fs) were only observed under R and B light (Fig. 3), but not under R7:B3 and WFL light. Perhaps this is because under the R7:B3 and WFL light, the horizontal bridges (hb) formed so rapidly, and the filamentous structures (fs) were formed at a stage earlier than 5 DAG. The plasmodesmata were evident in the grafting union between rootstock and scion on the 5th day after grafting under R7:B3 light (Fig. 6a–c). Magnification of the observed interconnections between tomato rootstock and scion showed the accumulation of apparent lipid or waxy material on their surface under blue light (Fig. 6d–f).

### The anatomical structure of the graft union at 8 DAG

**Figure 4:**
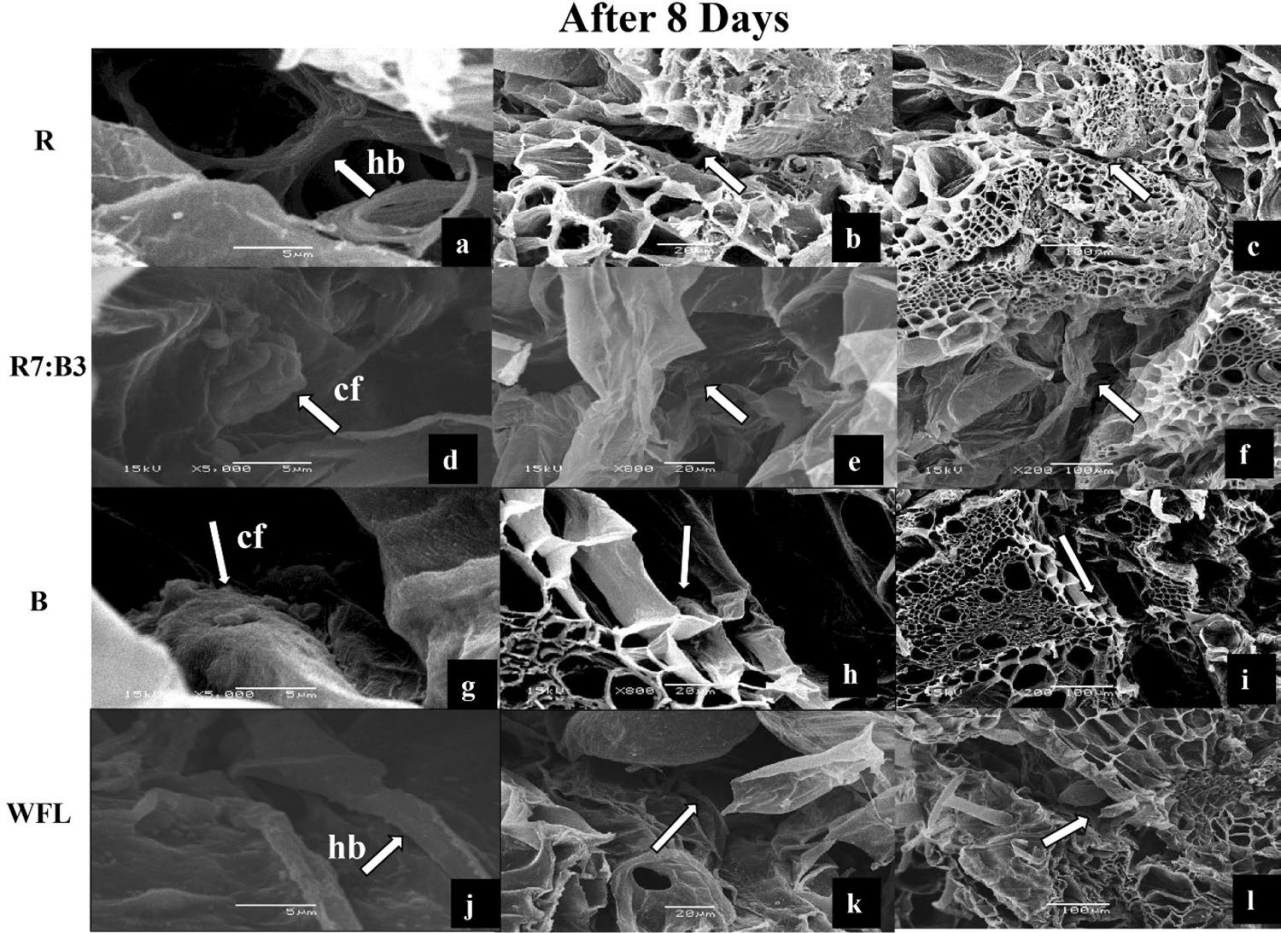
SEM images of interconnections between rootstock and scion after 8 days under R light (**a**–**c**), R7:B3 light (**d**–**f**), B light (**g**–**i**), and WFL light (**j**–**l**) light at the 8th days after grafting. fs—filamentous structures; hb—horizontal bridge; cf — formation of callus tissue.

In the images of SEM, during the second term of the study (8 DAG) (Fig. 2b,f,j,n), some horizontal bridges (hb) were observed between the rootstock and the scion before the formation of callus under the R light (Fig. 4a–c), while the horizontal bridges (hb) were observed to form after the 5th day under the WFL light, the single thickness was increased, but the callus was not formed (Fig. 4j–l). Moreover, the formation of callus tissue (cf) between the rootstock and scion was observed under R7:B3 (Fig. 4d–f) and B light (Fig. 4g–i). It is worth noting that grafting occurred under B light. Although the formation of horizontal bridges (hb) was delayed until day 5 (Fig. 3g–i), callus formed early until day 8. Day. This could be due to the importance of blue light in callus formation (Fig. 4g–i).

### The anatomical structure of the graft union at 11 DAG

**Figure 5:**
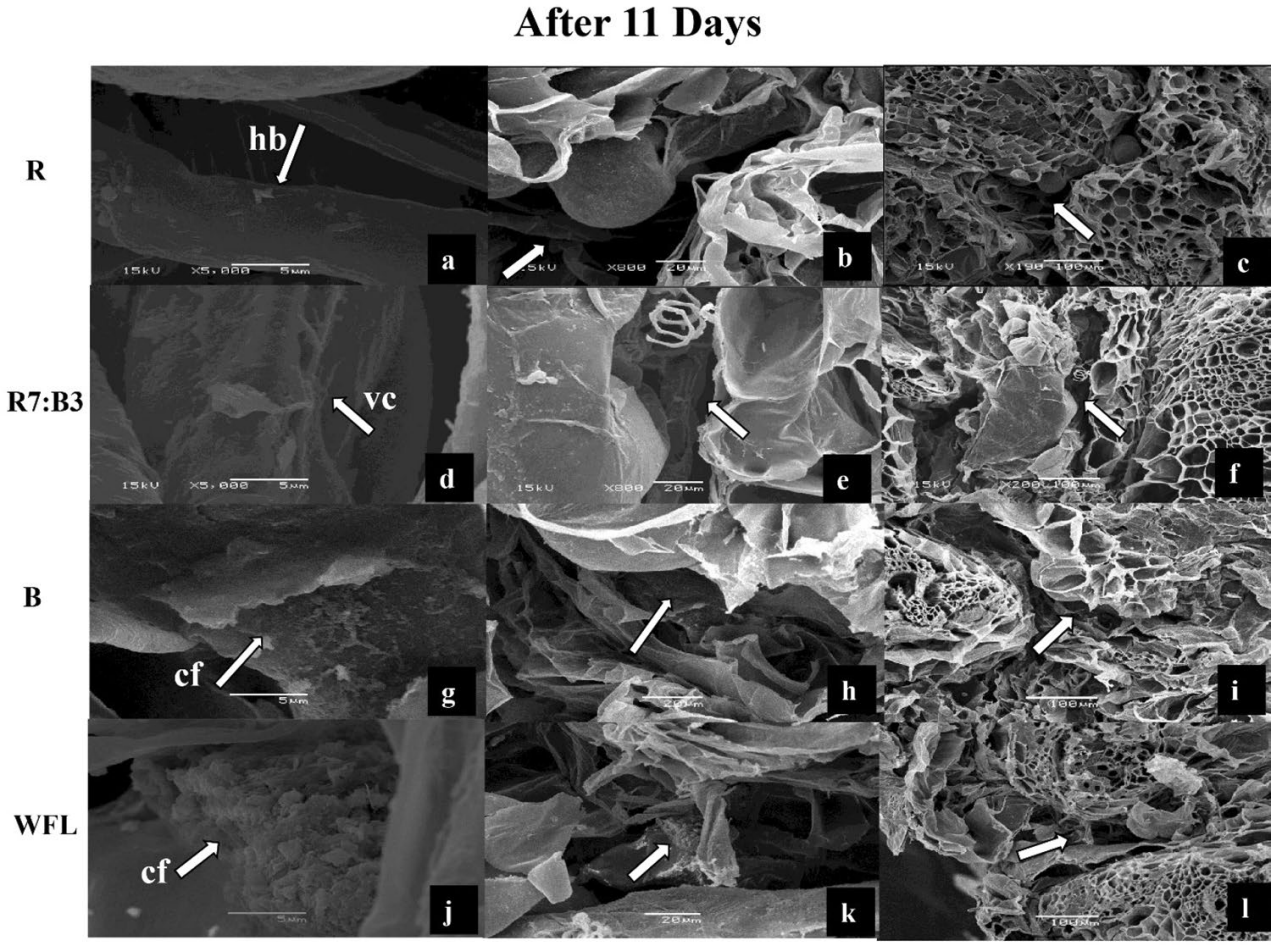
SEM images of interconnections between rootstock and scion after 11 days under R light (**a**–**c**), R7:B3 light (**d**–**f**), B light (**g**–**i**), and WFL light (**j**–**l**) light at 11th days after grafting. hb—horizontal bridge; cf—formation of callus tissue; vc—vascular connections.

In the SEM images, during the third appointment of the examination (11 DAG) (Fig. 2c,g,k,o), cell division and differentiation continued. However, different cellular behaviour occurred between treatments as the graft developed. The horizontal bridges (hb) were observed between the rootstock, and the scion under R light, the thickness of horizontal bridges was increased, but the callus was not formed (Fig. 5a–c), while the formation of callus tissue (cf) was continued till 11th day under the B light (Fig. 5g–i) and WFL light (Fig. 5j–l). In addition, under R7:B3, the cell division and differentiation continued, and vascular connections (vc) were established (Fig. 5d–f).

### The expression of genes related to auxin hormone pathway

To better understand the developmental processes occurring at the graft intersection and the extent to which the light spectra influence the success of the grafting process, we examined the genes expression by qRT-PCR from RNA libraries from tomato hypocotyl in the graft unions at 0, 3, 7, 11, and 20 days (Fig. 2d,h,l,p) after grafting in biological replicates for each tissue at each time point. We looked at the expression of markers associated with the vascular formation and cell division. Different artificial light sources influence the genes related to the Auxin hormones' signaling pathways during grafting process at different time points. As shown in Fig. 7, the expression level of the SAUR67, AUX1, and ARF30 genes were up-regulated at all different time points days after grafting, while the expression level of the LAX3 gene was down-regulated at all different time points after grafting. The expression level of the SAUR67, AUX1, and ARF30 genes slowly decreased with continued time points, suggesting that these genes play an essential role from the beginning of the grafting stages to encourage healing in the grafting process. The expression level of the SAUR67, AUX1, ARF30, and LAX3 genes were highest under R7:B3 at 3, 7, 11, and 20 days after grafting (Fig. 7a–d), respectively. The relative expression of SAUR67, AUX1, and ARF30 genes in grafted unions were (5.14, 2.71, and 20.64)-fold at 3 days after grafting, respectively, while the relative expression of LAX3 gene in grafted unions was (− 1.008)-fold at 3 days after grafting under R7:B3. There were apparent differences between the treatments in their effect on gene expression, which suggests that the expression of the SAUR67, AUX1, ARF30, and LAX3 genes were affected by the light spectras' different graft-healing process.

## Discussion

It is complicated to ensure successful grafting because of the complexity of the physicochemical processes involved in forming the graft union. This process relies on identifying root formation, which promotes the rapid development of vascular connections between the root and scion and the resumption of root and scion^28^. In the first phase of graft union formation, the intimate contact of the graft tissues of root and scion stem is brought together, and the parenchyma cells begin to regenerate to interlock, then root and scion stem. The second phase is the phase of cell differentiation, in which the cells begin to differentiate due to the original intimate contact. In the third phase, the graft union formation reaches on to the final form, forming a new convex layer between the callus bridges^26,27,29,30^. A study was conducted to evaluate the graft union formation of artichoke grafted onto two cultivated and wild cardoon rootstocks showed union bridges between rootstock and scion after three grafting days. After the appearance of connecting tissues, callus development was observed in the fall season. While in the spring season, the joints appeared after 6 days of grafting, indicating the significant role of environmental conditions on graft union^27^.

An anatomical study of the graft union formation in tomato showed that the connecting tissues between the scion and the rootstock appeared after 8 days of grafting, while callus formation and vascular rearrangements failed to connect them. The bridges of vascular bundles appeared on the 11th day after grafting, and the connection between rootstock and scion was found. The graft union formation took 7–14 days depending on the environment^26^.

**Figure 6:**
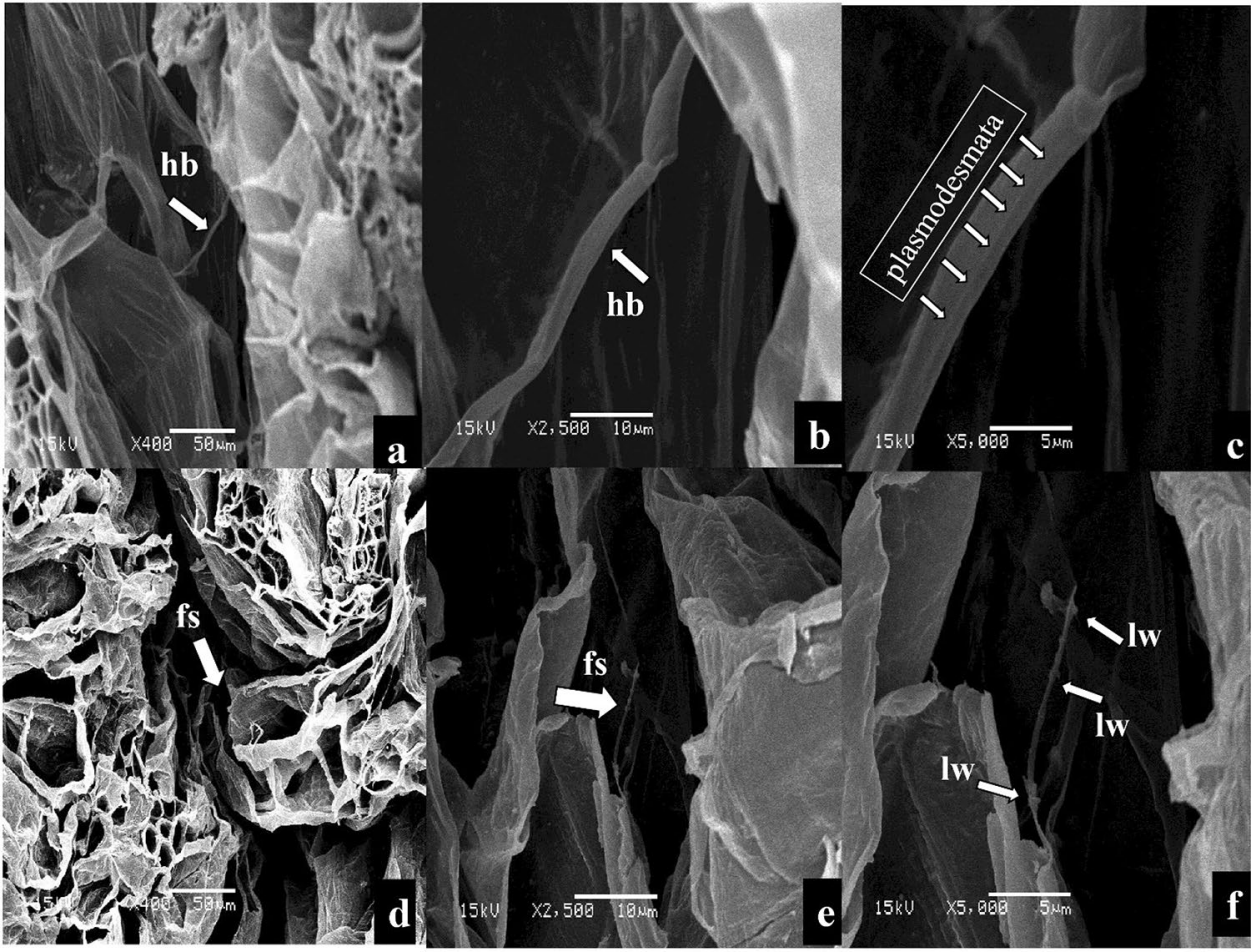
SEM images of interconnections in union grafting under R7:B3 light (**a**–**c**) and B light (**d**–**f**) grafting combinations, after 5 DAG. Magnification shows the plasmodesmata under R7:B3 (**c**), the structural features of filamentous interconnections, and the entity of lipid/waxy materials on their surface under BED light (**f**). fs—filamentous structures; hb—horizontal bridge; lw—lipid/waxy.

We assume that grafting is effective and complete only when some phloem and xylem connections across the matrix occur through cytoplasmic cell assemblies called plasmodesmata^28,31–35^. In general, plasmodesmata are involved in the symplastic transport of materials between cells in living plants. During the formation process, the callus' expansion, as an undifferentiated fuzzy mass formed by delicate walled parenchyma cells, precedes the development of plasmodesmata in the callus tissue to allow symplastic transport^31,36^. The cell wall complexes, representing the graft interface are non-division walls that originally consisted of the walls belonging to opposing cells and then fused. Pina et al.^36^ demonstrated the role of useful plasmodesmata at the joining interface in this way and showed strong species- and formation-related differences in the conductivity of plasmodesmata. After-effects of analyses involving developmental proteins show that the propagation of the calming signal, in any case, depends mainly on the size of the plasmodesmata hole^37^. Recent reports have uncovered that the genomes including nuclear, mitochondrial, and chloroplast genomes of rootstock and scion are combined at the graft site to generate cells with genomes from both graft guards^38–42^. We found that the accumulation of apparent lipid or waxy material on their surface under B light between the joints of rootstock and scion (Fig. 6f), Trinchera et al.^27^ suggested that could act as a preservative cover to resist mechanical stress. The results showed that R7:B3 and B-light should be considered as the most promising grafting combination.

We found that callus formation stages differ with the light at 8 DAG (Fig. 4) and 11 DAG (Fig. 5). Some authors confirmed that a mixture of red and blue light significantly affects callus growth and formation in vitro. Le and Tanaka^43^ reported that callus proliferation was best under 75% red LEDs + 25% blue LEDs at 45 μmol m^−2^ s^−1^ with 16-h photoperiod. Nhut has proved that a mixture of light (60% red LED and 40% blue LED) had a significant effect on callus growth during somatic embryogenesis of *Panax vietnamensis* Ha et Grushv., but it was not better than yellow light^44^. The utilization of blue LED lights on in vitro callus cultures of *Gynura procumbens* (Lour.) Merr induced a high antioxidant activity and enhanced the accumulation of total phenol content (TPC), total flavonoid content (TFC), and total anthocyanins TAC^45^. The red-light condition is strong of the most significant biomass aggregation, while violet light condition invigorated the most extreme phenolic and flavonoid blend in callus culture. The lower SOD and POD levels and MDA content in callus, developed in red light condition were normal for higher biomass arrangement Adil et al.^46^.

**Figure 7:**
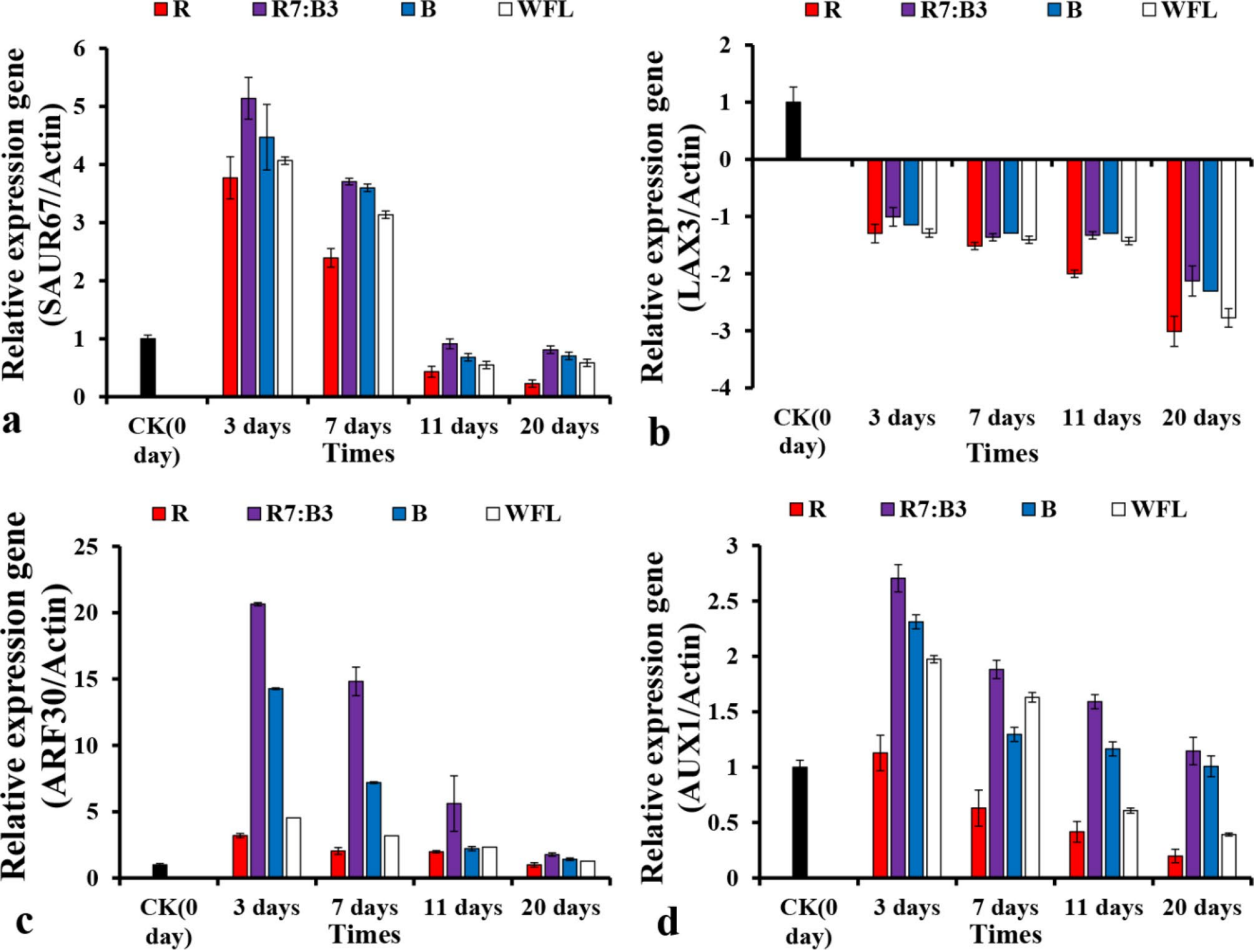
Relative expression of (SAUR67) (**a**); Relative expression of (AUX1) (**b**); Relative expression of (ARF30) (**c**); and Relative expression of (LAX3) (**d**) at different time points. The different letters show significant differences among light treatments according to the Duncan test (P < 0.05).

The present RT-qPCR analysis showed changes in the expression levels of genes associated with auxin signaling; the SAUR67, AUX1, and ARF30 genes were up-regulated at all different time points days after grafting, while the expression level of the LAX3 gene was down-regulated at all different time points after grafting (Fig. 7). This result is consistent with the RT-qPCR analysis of homo-grafting and hetero-grafting in tomato seedlings^20^. Because SAUR up-regulation is sufficient to induce growth^47,48^, other upstream factors may regulate SAUR-mediated growth independent of the auxin pathway. SAURs have thus been unveiled as growth-factors that are essential for both normal plant development and adaptation to environmental conditions. The up-regulation of SAUR genes can induce cell elongation in Arabidopsis^49–51^. Up-regulation of ARF during grafting further regulates various biochemical pathways promoting a vascular connection between the scion and the stock^52^. The expression pattern in most LAX genes during graft development is down-regulated^53^.

In conclusion, our results indicate that the vascular connections between the root and scion stocks appeared on the 11th day after grafting in the plants treated with R7:B3 to ensure grafted tomato plants' survival. However, further studies are needed to investigate these results for graft development in tomato plants.

## Materials and methods

### Plant material and culture conditions

**Figure 8:**
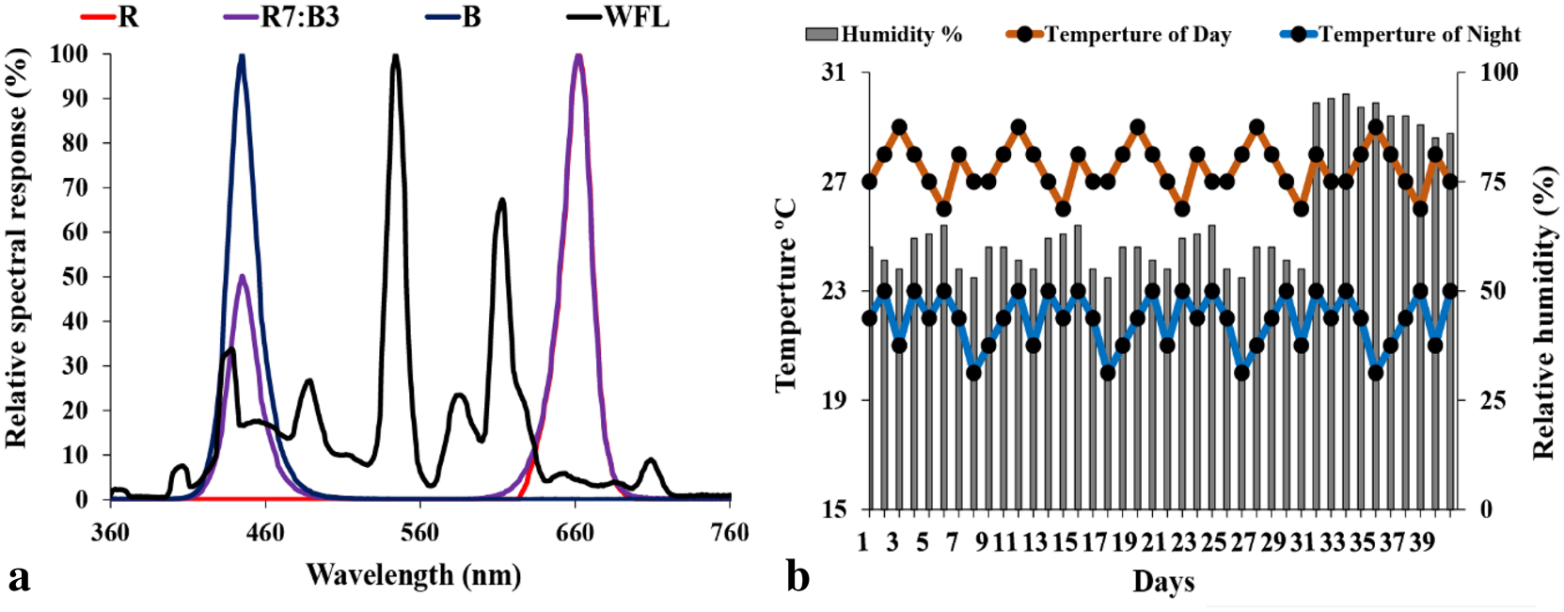
Spectrum distribution of the treatments LED light in the experiment (**a**) and Environmental conditions (**b**). Where R = Red light 100%, R7:B3 = Red70% + Blue30%, B = Blue light 100%, WFL = White Fluorescent Lamps100%.

Rootstock cultivar of tomato (*Solanum lycopersicum* L. Cv. Gangmu No.1) was resistant to bacterial wilt brought from the company;—Kaikai 1681 Seeds (Weifang, Shandong Province, China) Co., Ltd. While a high yielding cultivar (*Solanum lycopersicum* L. Cv. Millennium) was selected as scion brought from the company;—Farmers' Friends Seedling (China) Co., Ltd. Our studies were complied with local and national regulations and following Fujian Agriculture and Forestry University (Fujian, China) regulations. The LED lamps (UH-BLDT0510) used in this study were manufactured by Kedao Technology Corporation (Huizhou, China). Tomato seeds were planted 30 days before grafting under 100 μmol m^−2^ s^−1^ R7:B3 (red ~ 70: blue ~ 30), which was chosen as the best LED source in our previous research^54,55^. The seeds were sown in trays (W 28 cm × L 54 cm × H 5 cm, Luoxi Plastic Products Co., Shandong, China) filled with the commercial growing substrate (N_1_:P_:1_K_1_ ≥ 3%, Organic matter ≥ 45%, pH 5.5–6.5, Jiangping Enterprise Co., Fujian, China). Environmental conditions in growth chambers are shown in Table 1 and Fig. 8. The average relative humidity varied in the first 30 days 55 ± 5%, and in the post-grafting period was 95 ± 5% (Fig. 8). Irrigation was provided to the seedlings daily. After 1 week of sowing, water-soluble compound fertilizers i.e., ("N_20_: P_20_: K_20_ + TE", Ruierkang Co., Russia, and Stimufol Amino (compound fertilizers "N 25%, P 16%, K 12%, Amino acids 2%, B 0.044%, Fe 0.17%, Mo0.001%, Zn 0.03%, Cu 0.085, Co 0.01%, Mg 0.02%, Mn 0.085% and EDTA" Shoura Co., Egypt.) were applied to the seedlings twice per week through irrigation. The grafted tomato seedlings were divided into three groups and placed in artificial climate boxes under 100 μmol m^−2^ s^−1^, R: red LEDs (662 nm), R7:B3: (red ~ 70%: blue ~ 30%; 662 nm, 445 nm), B: blue (445 nm), and WFL: white fluorescent light (control) (544 nm) for 12 h per day.

**Table 1:** The primer sequences of genes related to the Auxin hormone signaling pathway.

No	Code	Gene	Forward primer (5ʹ–3ʹ)	Reverse primer (5ʹ–3ʹ)	KEGG: annotation
1	SAUR63	Solyc07g014620.1.1	TGGGTATCAAAGAACCGGCG	CGGTCTTCATCCCGGTTCAA	Auxin-induced SAUR-like protein SAUR family protein
2	AUX1	Solyc11g013310.1.1	GGGTGGGCAAGTATGGTCAA	CAAGCCTTATGTGGAGGGCA	AUX1, LAX; auxin influx carrier (AUX1 LAX family)
3	ARF30	Solyc01g108350.2	ACTACATGGATTGGGCTCGC	CTCCATTTGCGTTTGGTGCA	Auxin response factor 30
4	LAX3	Solyc01g111310.2	GGTTGGGCCAGTATGACCAA	GCACCAACATGAGGGAGGAA	Auxin transporter-like protein 3 (LAX3), mRNA

### Grafting experiment

The experiment was designed under a completely randomized design with three replicates. The rootstock and scion were splice-grafted after 30 days of sowing (3–4 compound leaves)^56^. The grafted seedlings were put in a box into LED chambers with high humidity of approximately 90–95% with a transparent cover on top. After 3 days, the ventilation holes were opened to avoid suffocation in the boxes. The transparent cover was removed for 5 min, and the period increases until the seventh day with high humidity of approximately 90%. After 7 days, the transparent cover was removed for 30 min daily, and humidity was adjusted at 70–90% approximately. After 9 days, the ventilation period was exceeded up to 2–3 h daily with humidity of approximately 70%. After 12 days, the union was completed between the rootstock and the scion^26^.

### Microscopic examination of anatomical structures

**Figure 9:**
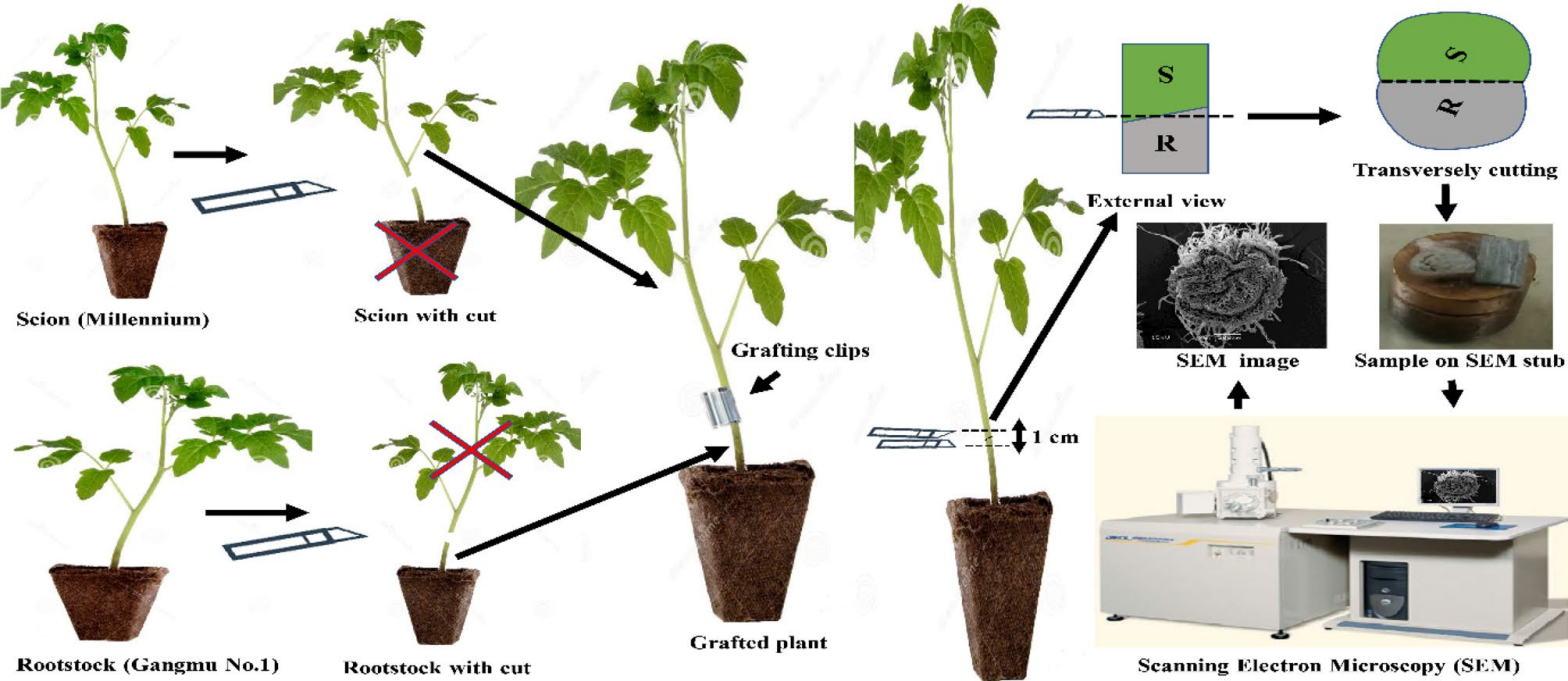
Illustration of the grafting procedure and preparing samples for examination with scanning electron microscopy (SEM).

The anatomical examination was conducted through scanning electron microscopy (SEM) to compare the plant tissues affected by LED treatments during graft union formation. The 1 cm precisely sectioned rootstock/scion samples from different LED treatments were obtained and used for imaging experiments. The transverse section of the graft union was cut through a sharp razor blade to check the tissue affinity among all treatments over time under SEM (Fig. 9). The imaging analyses were performed on the 5th, 8th, and 11th days after grafting (DAG) to evaluate the matrixs' wound healing process. Each treatment was evaluated in three replications to ensure the results.

SEM images were obtained using the JSM-6380 microscope (JEOL, Ltd., Tokyo, Japan), resolution 3.0 nm (30 kV, Working Distance WD 8 mm, SEI detection modes), accelerating voltage 0.5 to 30 kV, magnification 5× to 300,000×, stage rotation 360°. The JSM-6380 sample chamber can assess a sample of up to 6-inches in diameter. The sample diameter was ⁓ 5 mm; each tissue portion was placed on the metal disc covered by a thin adhesive layer (Fig. 9).

#### Sample processing procedures

Fixed the sample with 5% pentanal for 4 h, then washed the sample with phosphate buffer (three times, with an interval of 10–15 min between each pass). 2—Put the samples in 1% of Osmium acid is fixed for 4 h, then rinsed with distilled water (three times, every 10–15 min). 3—Put the samples in Ethanol for dehydration step by step; (50%, 70%, 80%, 90%, 100%, every 10–15 min interval, 100% replacement three times), then replaced Ethanol with Propylene oxide; (two time). 4—Critical point drying; (HITACHI HCP-2) was a process to remove liquid precisely and controlled way. 5—Put the samples on the stub, then sprayed gold (EIKO IB-5). 6—Observed and took photos on the machine^57^.

### Sample collection and RNA isolate

Samples were collected to study gene expressions from the graft union at different times after the grafting process. Thirty different clone samples of rootstock (about 5 mm) and scion (about 5 mm) from the graft unions were collected at 0, 3, 7, 11, and 20 days post-graft, the 0-day post-graft (5 mm rootstock + 5 mm scion from graft area before grafting process) was the external control. Subsequently, the samples were immediately frozen in liquid nitrogen and stored at − 80 °C. For RNA extraction, each frozen sample was ground to a fine powder in a stainless-steel grinder. Total RNA was isolated with TRIzol Reagent, following the manufacturers' protocol (RNAprep Pure Plant Plus Kit, Tian Biotech (Beijing) Co., Ltd). The RNA quality was assessed using electrophoresis on a 1.5% agarose gel. The total RNA concentration was determined by measuring the absorbance ratio (A260/280) ranging from 1.8 to 2.0 was used for quantitative real-time polymerase chain reaction (qRT-PCR) analysis on a Nanodrop (Thermo; Nanodrop 2000, USA).

#### cDNA synthesis

Total RNA samples of the experimental were reverse transcribed into cDNA using the PrimerScript reagent kit with gDNA Eraser (Perfect Real Time), following the manufacturer's instructions (Takara Bio USA, Inc.). The cDNA has been diluted to 2X by RNase free dH_2_o.

#### Quantitative real-time PCR analysis:

The transcript data for the *Solanum lycopersicum* genome (release ITAG2.4) retrieved from the JGI-sequenced plant genomes website (https://phytozome.jgi.doe.gov/pz/portal.html#!info?alias=Org_Slycopersicum). Expression of 4 DEGs (SAUR67, AUX1, ARF30, LAX3) and internal control gene (actin) were measured by relative real-time PCR analysis in a 96-well plate. The annealing temperature was between 59 and 60 °C for qRT-PCR. The amplification was performed in a 15 µL reaction volume containing 7.5 µL of TransStart Tip Green qPCR SuperMix, 0.3 µL of each primer, 5.9 µL of RNase free dH_2_O, and 1 µL of the template cDNA. The qRT-PCR was performed using Lightcycler96 software 1.1. The primer pairs used for the qRT-PCR quantification analysis were designed using Primer3Plus (https://primer3plus.com/cgi-bin/dev/primer3plus.cgi); the primer sequences are listed in Table 1. The PCR preincubation conditions were as follows: 95 °C for 30 s, The PCR amplification conditions were by 45 cycles of 95 °C for 5 s, and 60 °C for 10 s, the PCR melting conditions were as follows: 95 °C for 5 s, 65 °C for 1 min, and 95 °C for 1 s, the cooling conditions were as follows: 50 °C for 30 s. Fluorescent signals were collected at each polymerization step. Three biological replicates and three technical replicates were used per sample. The different gene expression was calculated by the 2^−ΔΔCT^ method^58^.

### Statistical analysis

The study was conducted under a completely randomized design (CRD) with three replicates. Expressed genes' data were subjected to one-way ANOVA using Duncan’s multiple range test (DMRT) method for pair-wise comparison of mean values at 5% significance level. SPSS statistical software package version 16.0 (SPSS Inc., Chicago, IL, USA) was used to analyze the data. Adobe Illustrator software package version 23.0.3 was used to improve the quality of the images.

## Conclusions

Our results showed that the quantity and the quality of the LEDs light have a significant effect on the structural development and matrix formation of grafted tomatoes. The grafted plants grown under the mixture of red and blue light in the ratio of R7:B3 had the faster cell division and proliferation processes. This in turn caused better matrix formation in comparison to all other light treatments/combinations. The expression of involved genes suggests that their up-regulation may contribute to the improvement of graft junctional arrangement of the grafted seedling. Although the understanding of matrix formation and grafting's structural development is complicated, our results partially revealed some new mechanisms behind successful grafting. Nevertheless, we still lack an understanding of callus formation in the grafting area. Further research should be conducted to explore the exact genomic mechanism behind the graft junction's development under the influence of LEDs.
